# Supplementary figures and images for: Quantitative analysis of rabies virus-based synaptic connectivity tracing
Source: PLoS One. 2023 Mar 30;18(3):e0278053. doi: 10.1371/journal.pone.0278053 (PMC10062636; doi:10.1371/journal.pone.0278053)

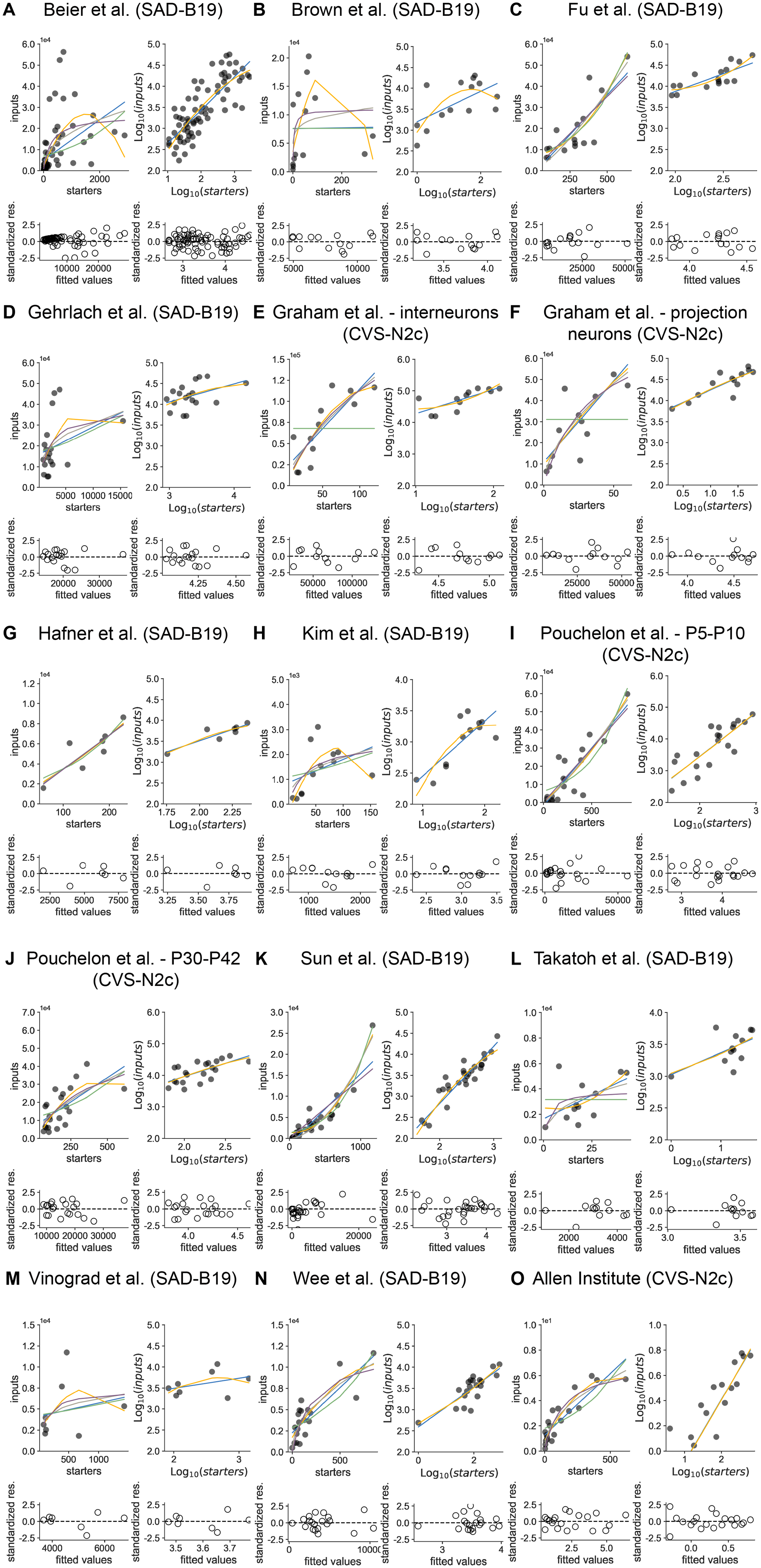

Supplement: S1 Fig — Colours indicate different fitted models, with the same colour-code as in Fig 1. Datasets are from [13–15, 18, 19, 21, 22, 25, 32, 36]. Please note that input quantification is done by counting labelled pixels instead of individual neurons in the Allen Institute dataset (panel O). (TIF) [file pone.0278053.s001.tif]

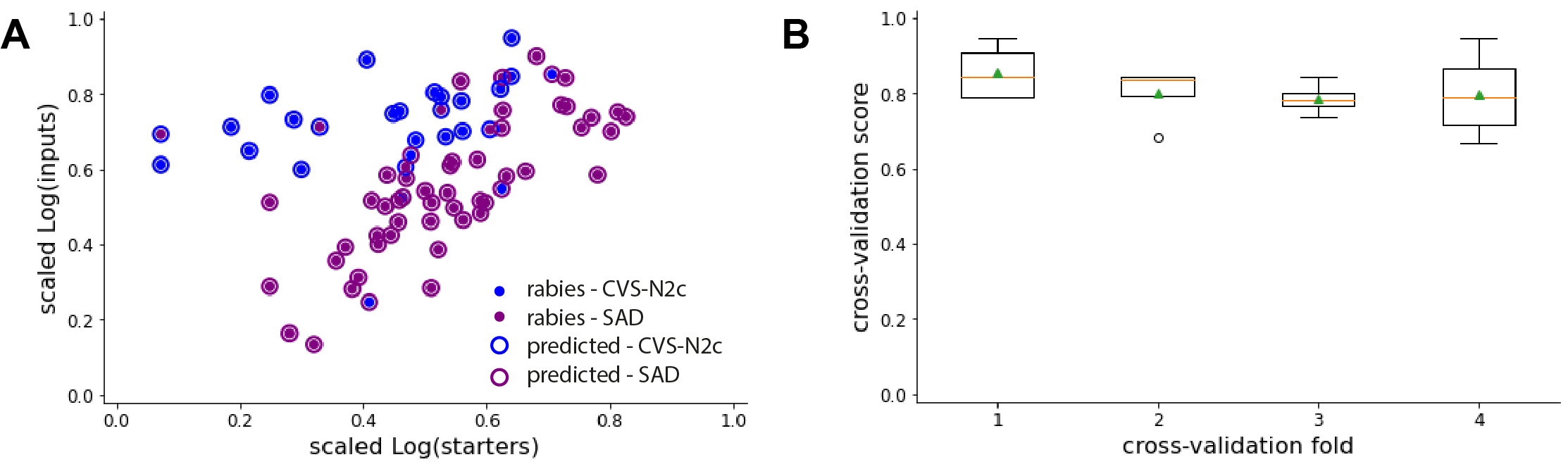

Supplement: S2 Fig — (A) Classification of the rabies strain used based on log(ns), log(ni) and starter cell type, for all pooled datasets where starter cell type was clearly identified as either pyramidal cells or interneurons and inputs quantified as cell counts. The model was a linear support vector classifier, and we used stratified 4-fold cross-validation to preserve the percentage of samples for each class. The plot corresponds to a single cross-validation fold. (B), Cross-validation scores show consistent accuracy across folds. (TIF) [file pone.0278053.s002.tif]

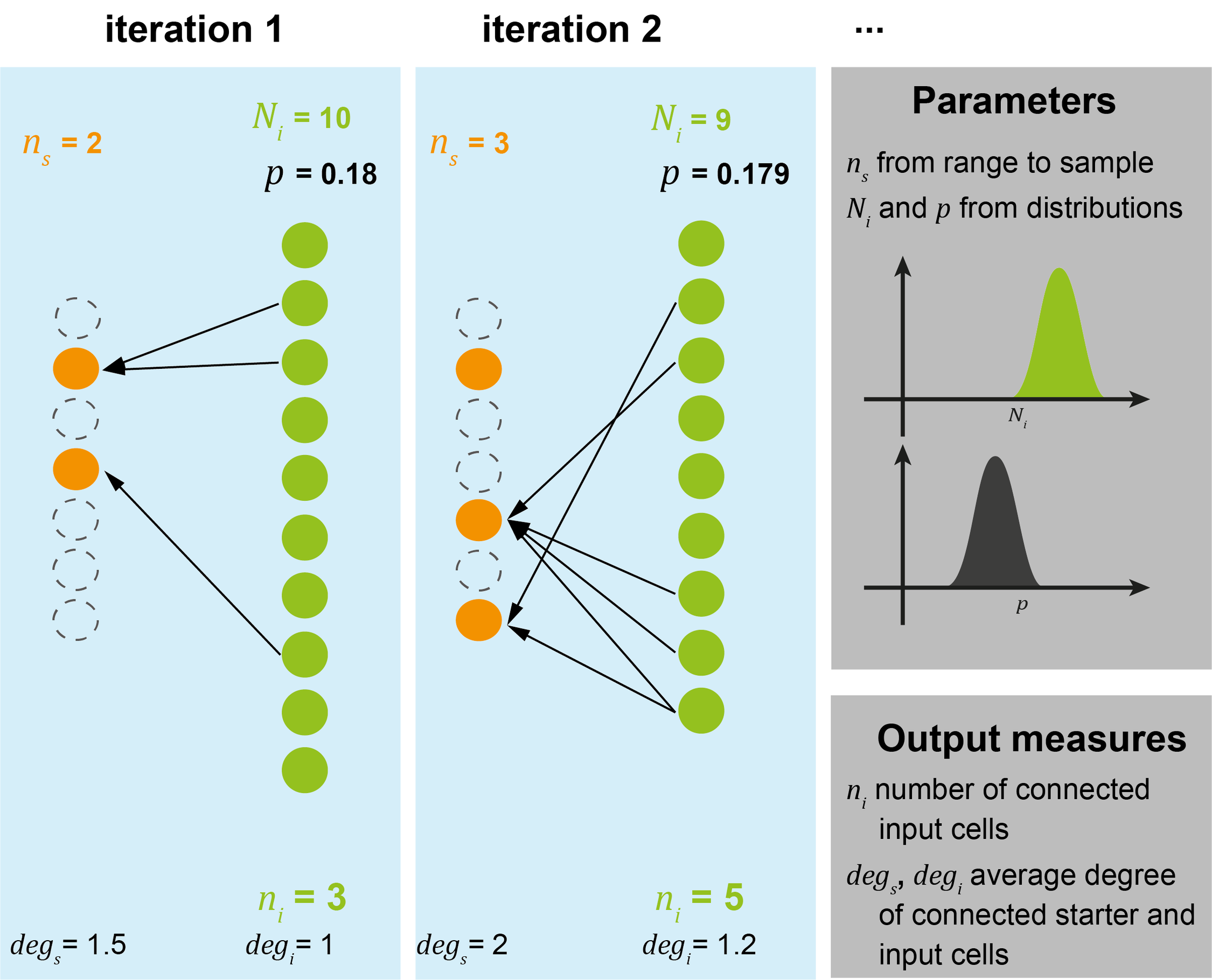

Supplement: S3 Fig — Illustration of input parameters, iteration steps and output measures for the probabilistic model. (TIF) [file pone.0278053.s003.tif]

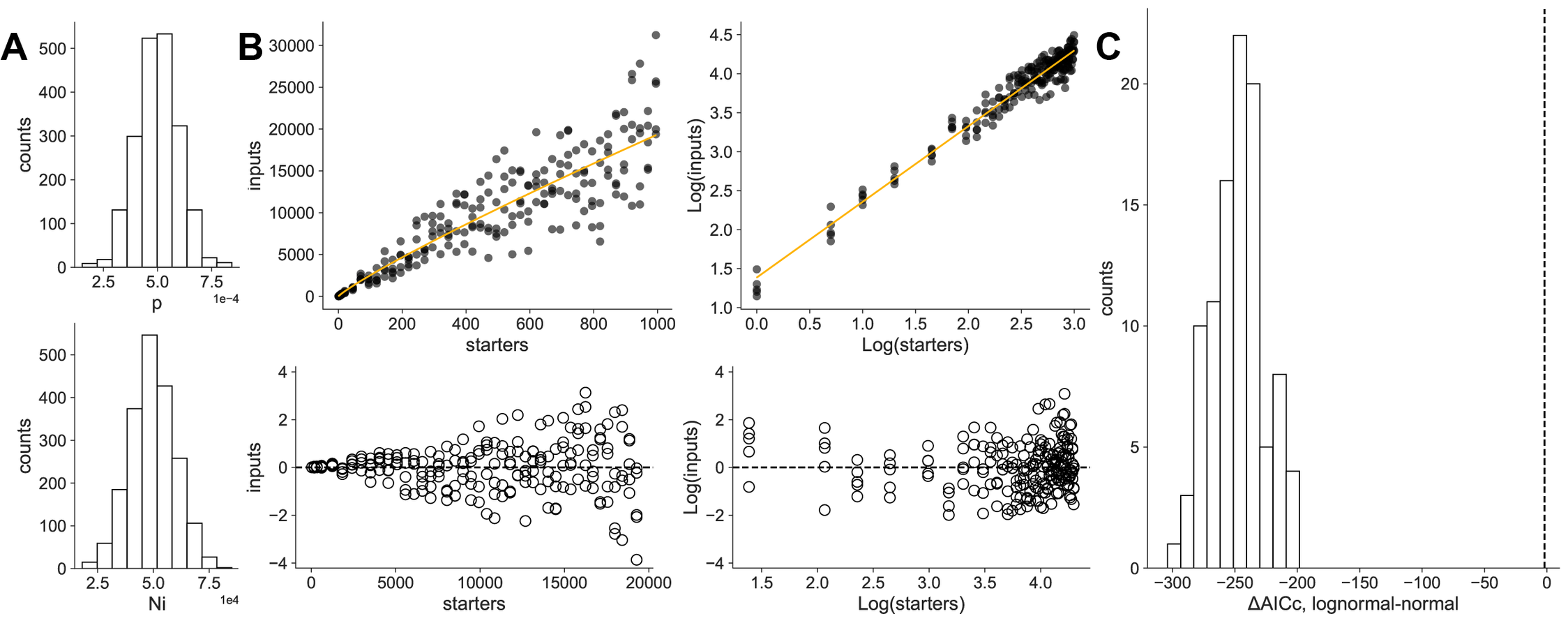

Supplement: S4 Fig — (A) Distributions of connectivity parameters (p, average 5*10−4, and Ni, average 50000 cells; both distributions have a s.d. of 0.2 * their average value). (B) Simulation of inter area connectivity with the probabilistic model plotted as in Fig 1. Connectivity parameters are randomly drawn from the distributions in A for each observation. (C) Simulations as in B were performed 100 times and residual analysis was performed for each resulting curve. Dotted line represents dAICc of -2. (TIF) [file pone.0278053.s004.tif]

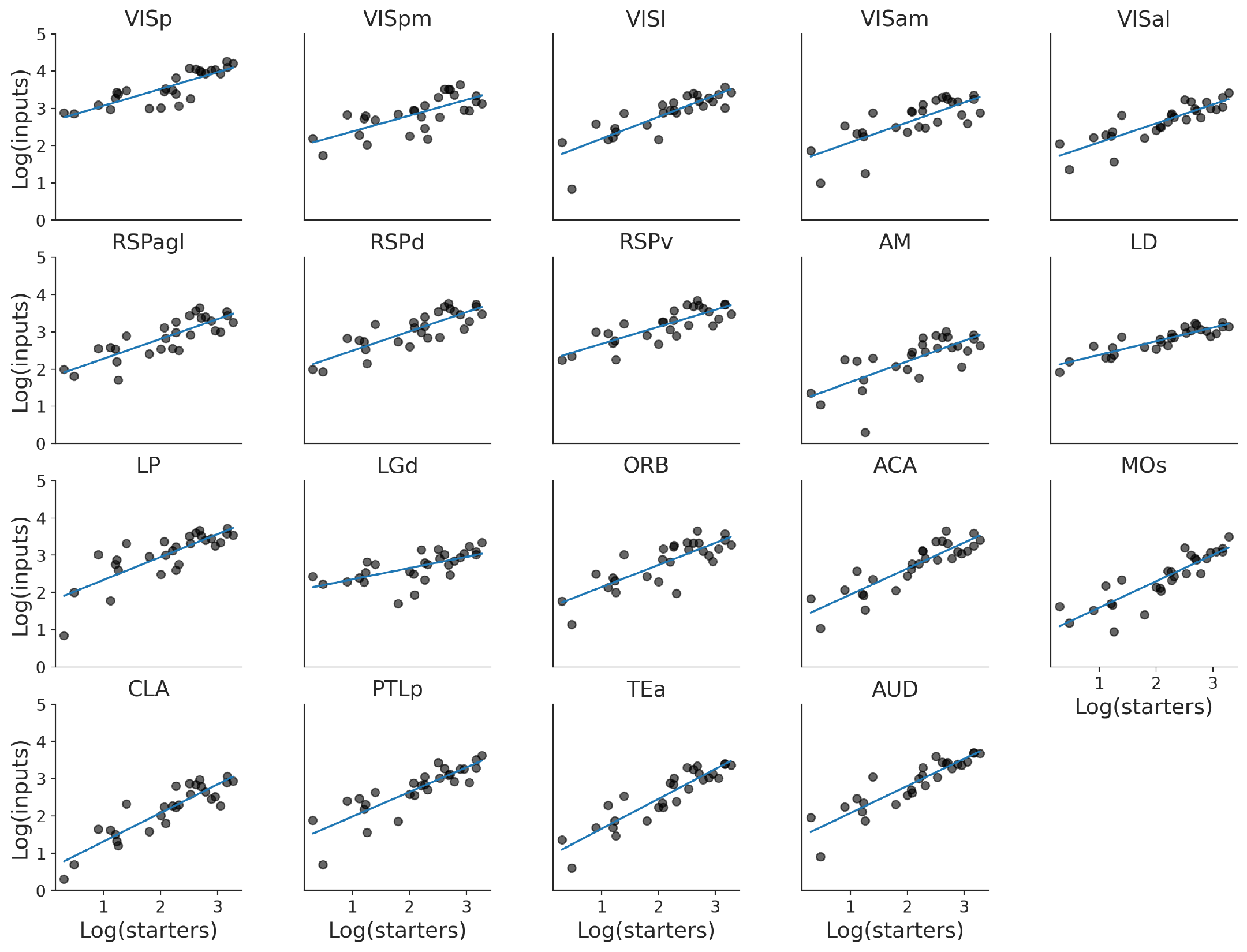

Supplement: S5 Fig — Linear fits of log-transformed ni vs ns relationship for individual brain areas. (TIF) [file pone.0278053.s005.tif]

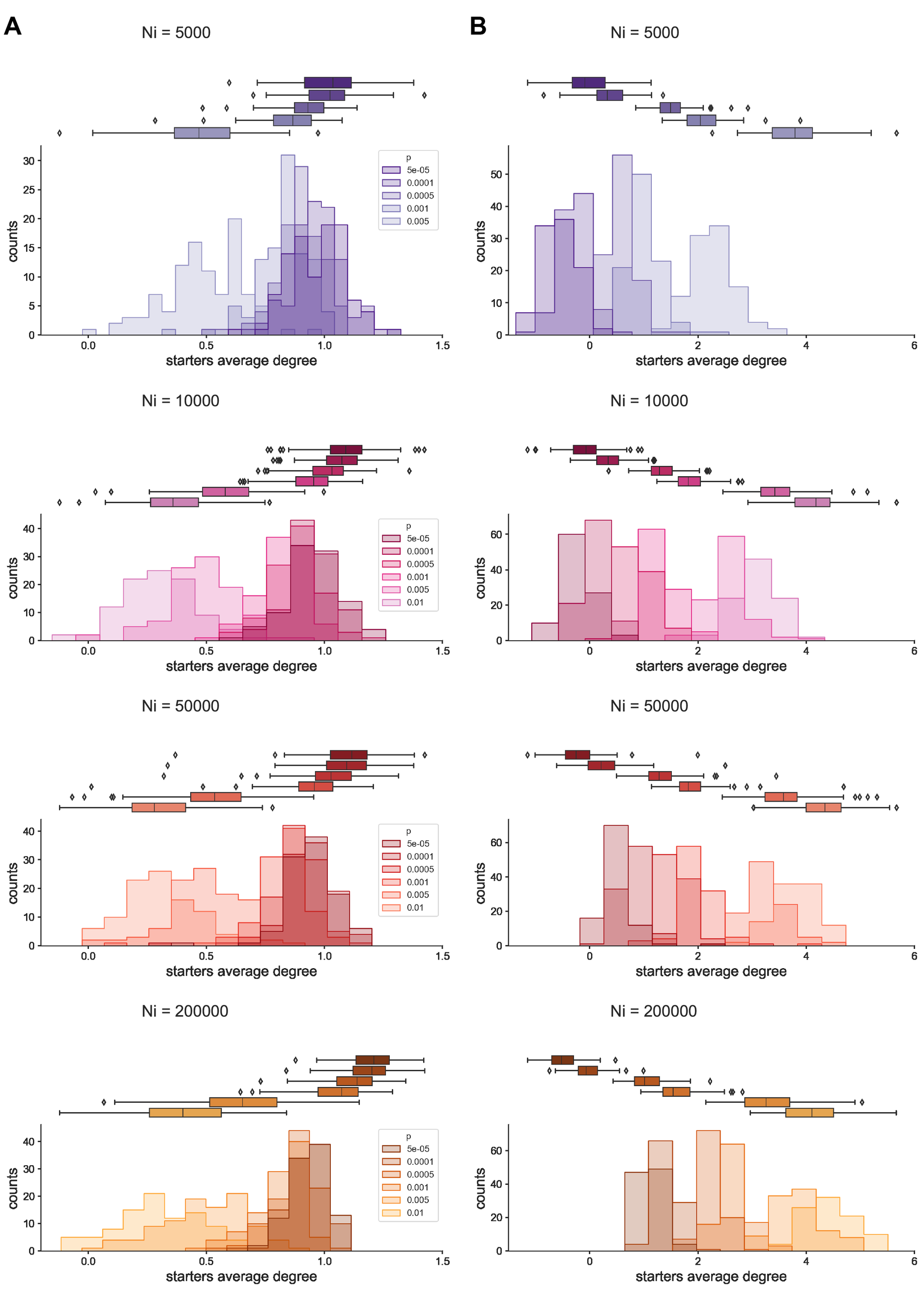

Supplement: S6 Fig — Distributions of slope (A) and y-intercept (B) values obtained across simulations with various model parameters (colours for Ni and shading for p), as plotted in Fig 2. Both Ni and p were drawn from distributions with a width of 0.2 * average. (TIF) [file pone.0278053.s006.tif]

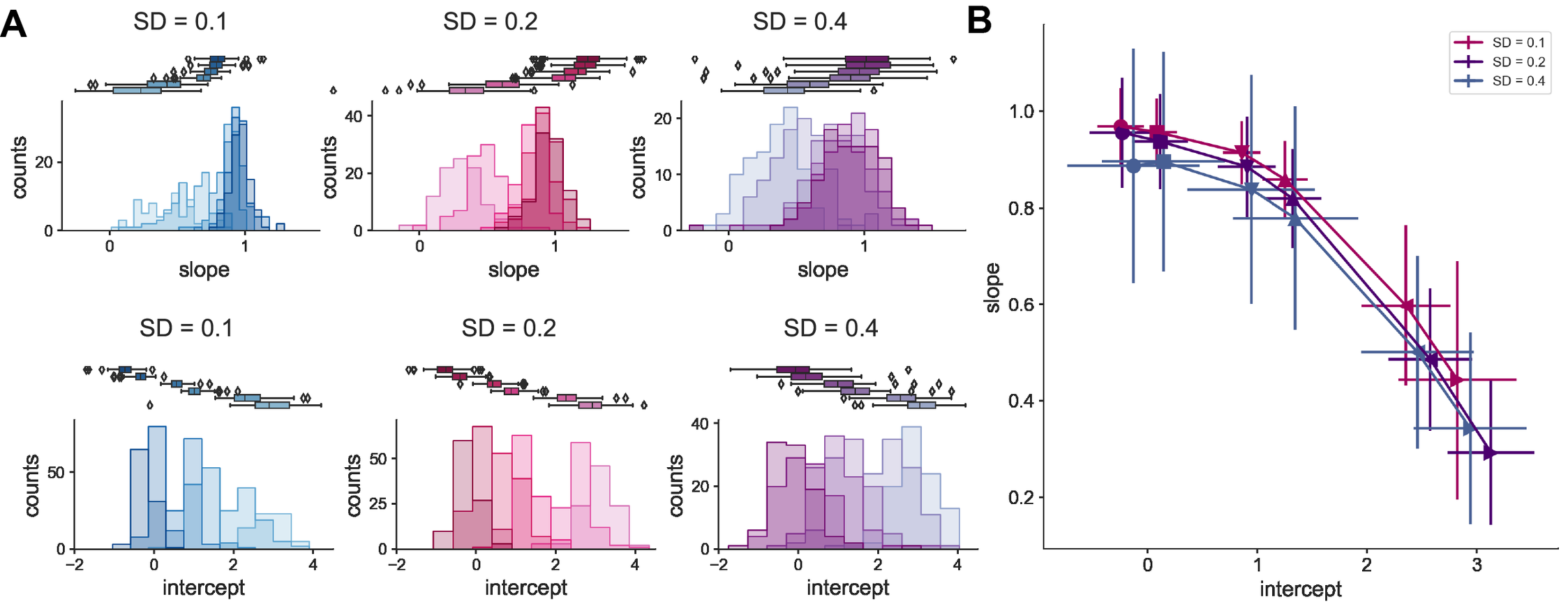

Supplement: S7 Fig — (A) Distributions of fit parameters of log(ni) vs log(ns) relationship for an average Ni = 10000, varying connection probabilities as in Fig 2 and parameters drawn from distribution of varying widths (S.D. = 0.1, 0.2 or 0.4 * average). (B) Slope vs y-intercept plot for an average Ni = 10000 with both model parameters drawn from distribution of varying widths. (TIF) [file pone.0278053.s007.tif]

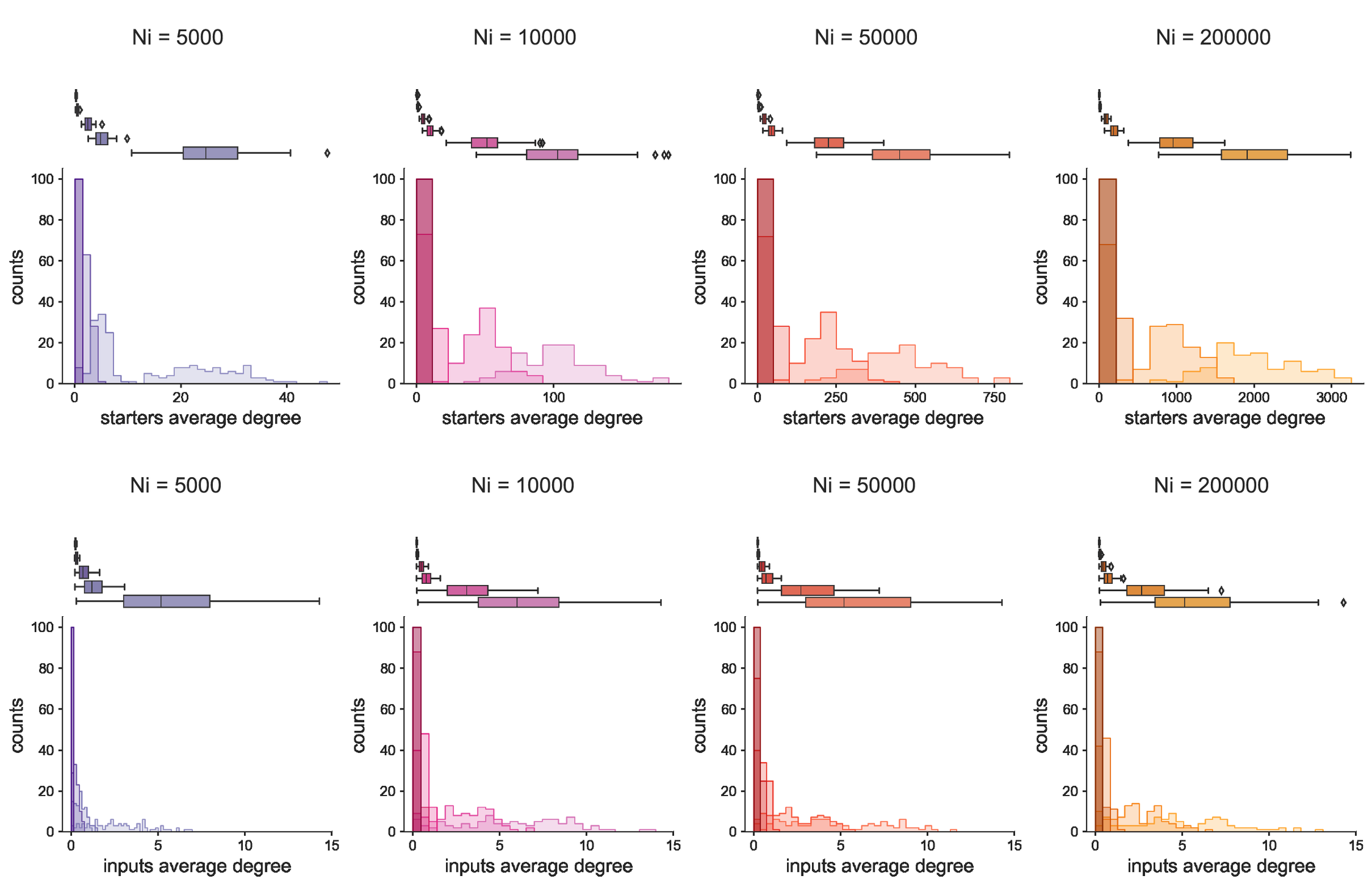

Supplement: S8 Fig — (Top) Distributions of starter cell degrees for varying Ni and p parameters. Both parameters were drawn from distributions of with 0.2 * average value of parameter. (Bottom) Distributions of input cell degrees for varying Ni and p parameters. Both parameters were drawn from distributions of with 0.2 * average value of parameter. (TIF) [file pone.0278053.s008.tif]

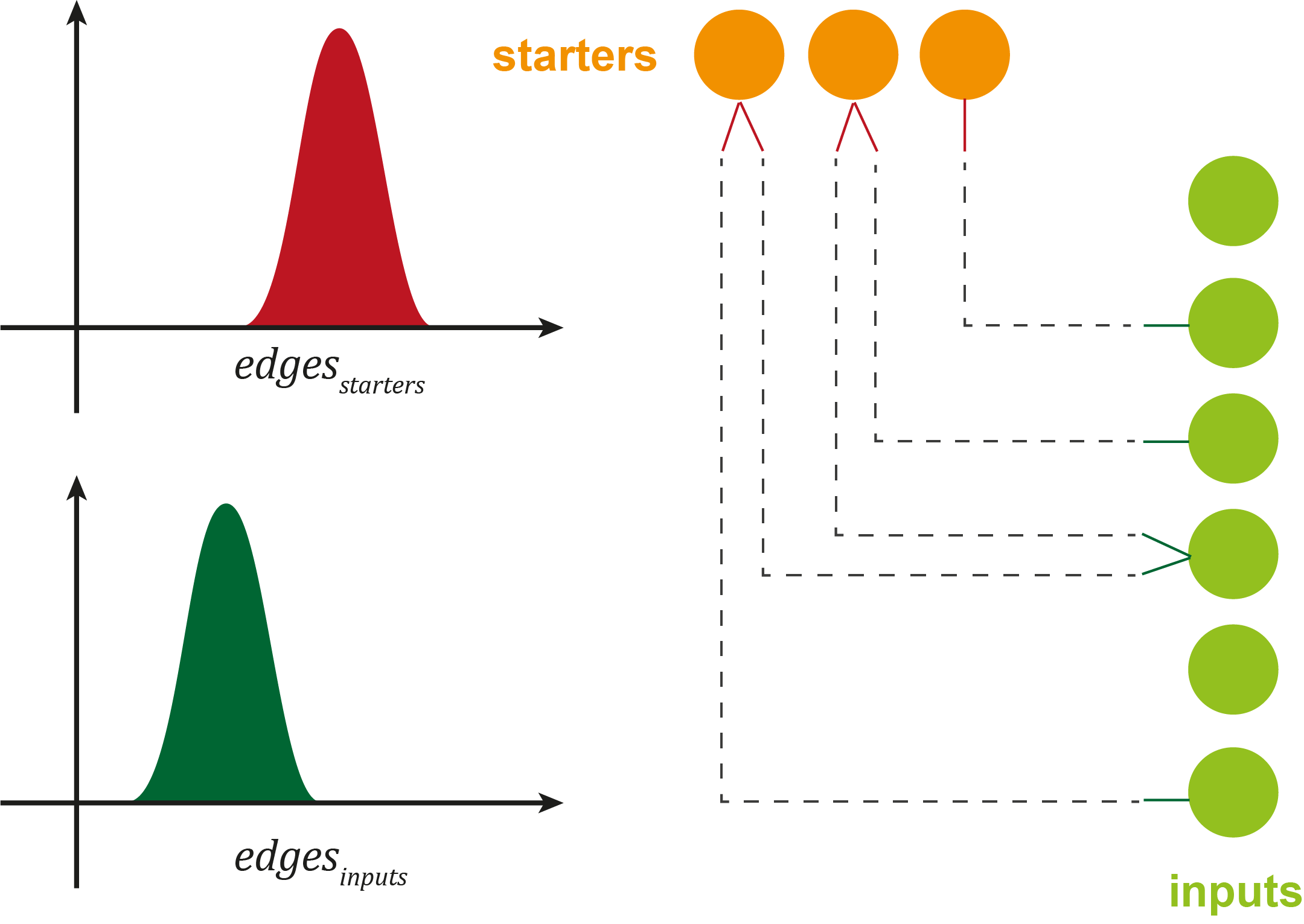

Supplement: S9 Fig — Illustration of a single step of the simulations for the configuration model. (TIF) [file pone.0278053.s009.tif]

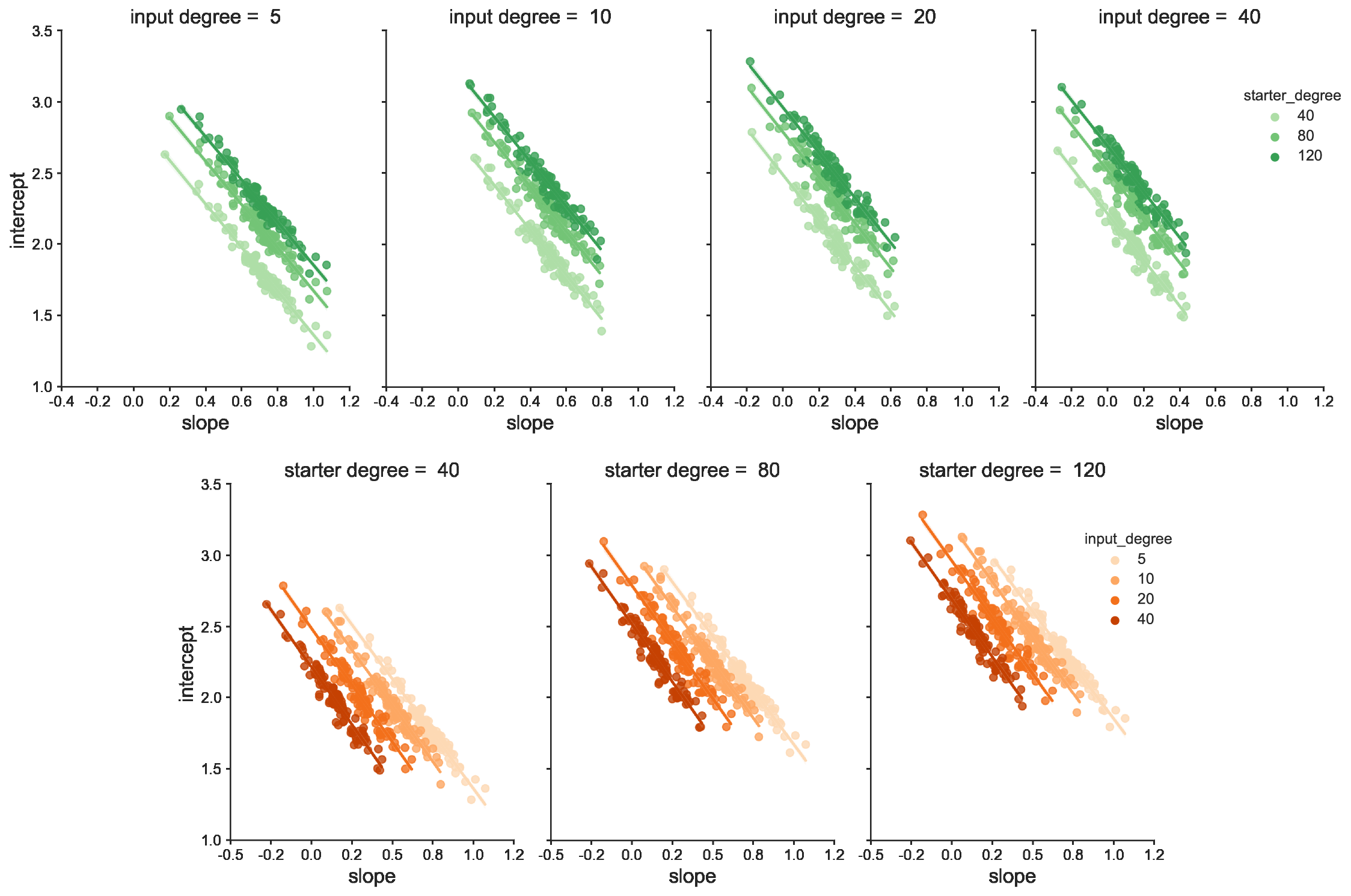

Supplement: S10 Fig — (Top) Influence of starter degree on intercept vs slope relationships (each panel is a specified mean input degree). (Bottom) Influence of input degree on intercept vs slope relationships (each panel is a specified mean starter degree). (TIF) [file pone.0278053.s010.tif]

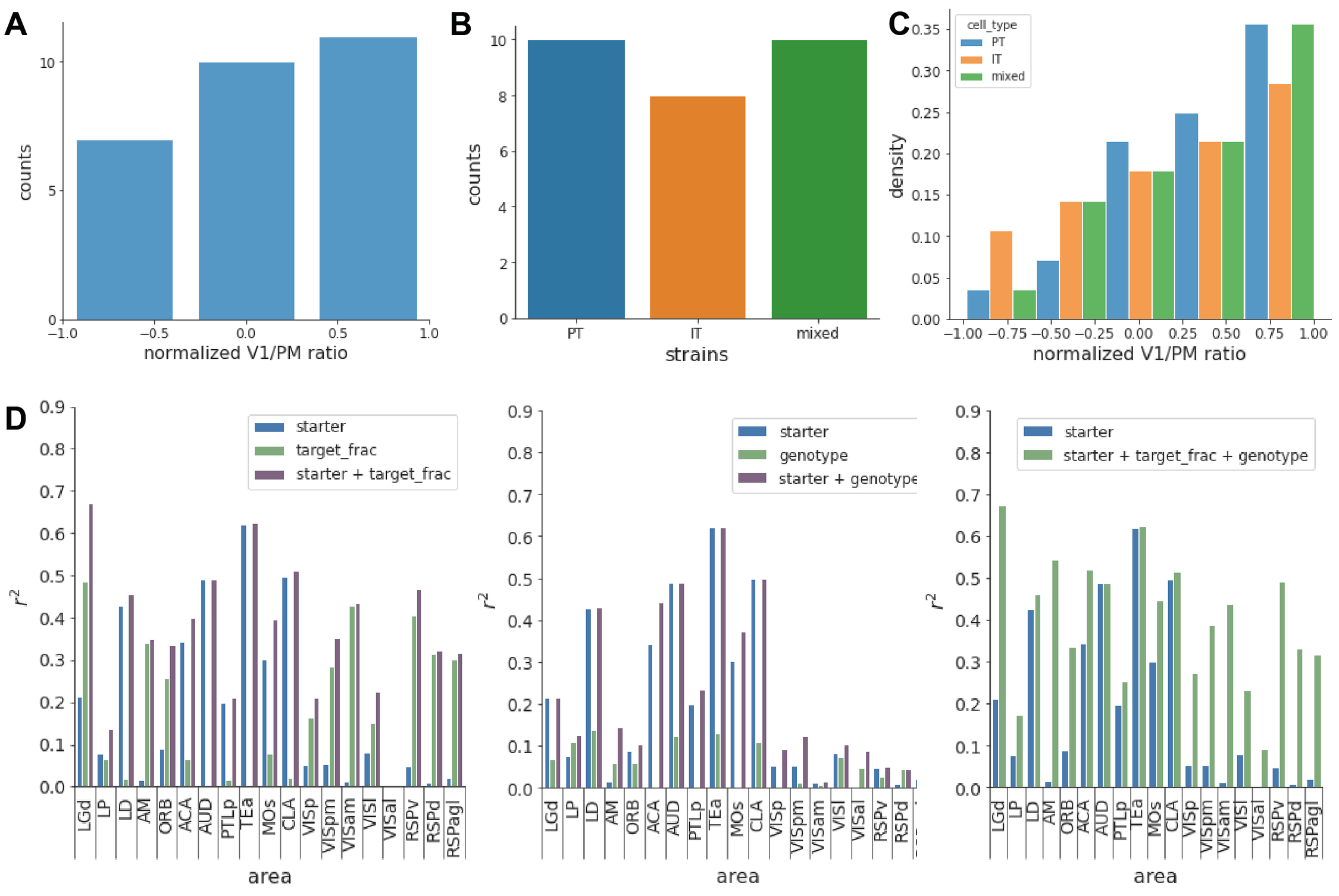

Supplement: S11 Fig — (TIF) [file pone.0278053.s011.tif]

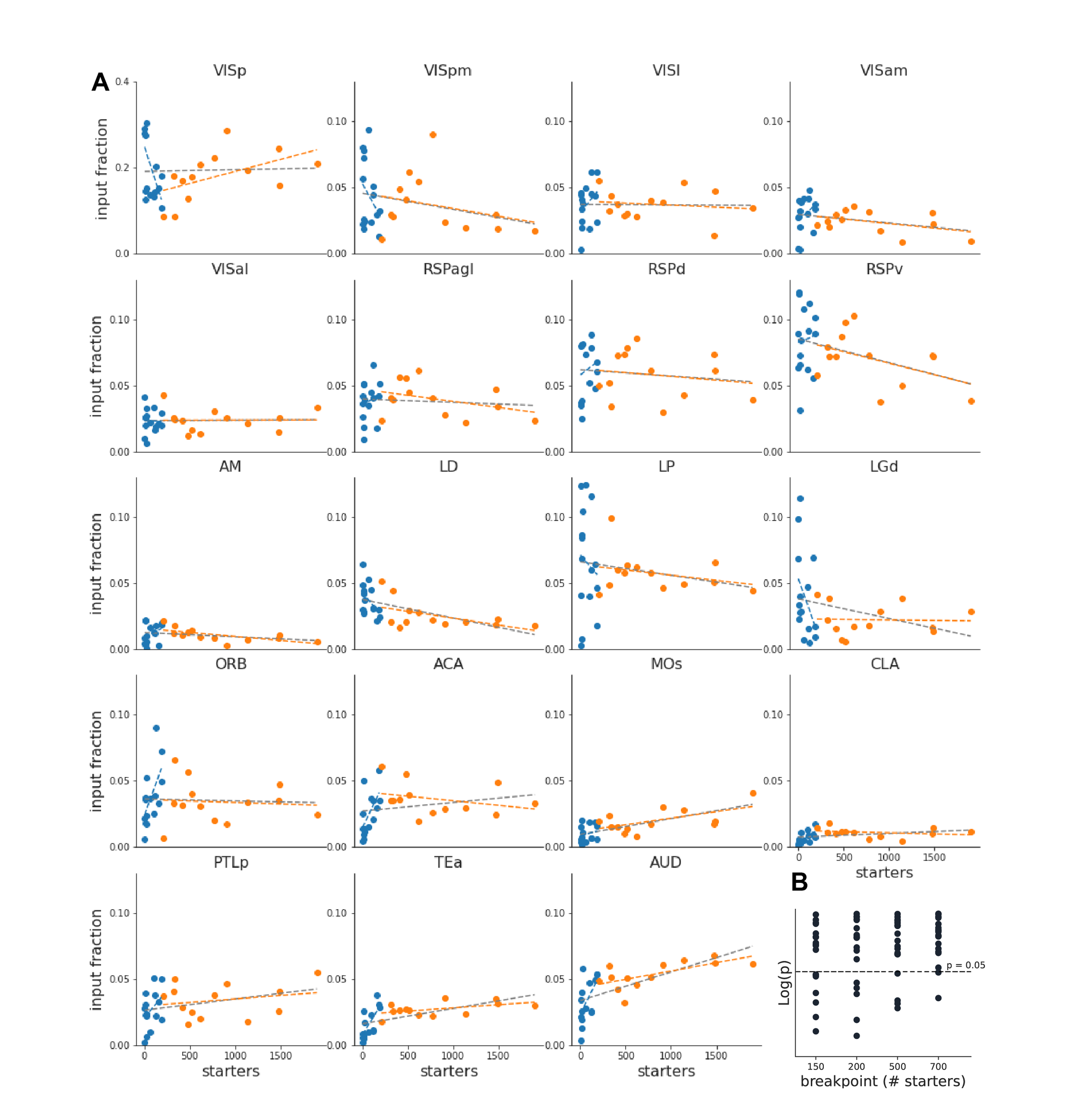

Supplement: S12 Fig — (A) Input fraction vs ns. Dashed lines represent linear fit through all data (grey), for ns < 200 (blue) or > 200 (orange). (B) p-value for Chow-test for varying break point values (x-axis), for individual brain areas. (TIF) [file pone.0278053.s012.tif]

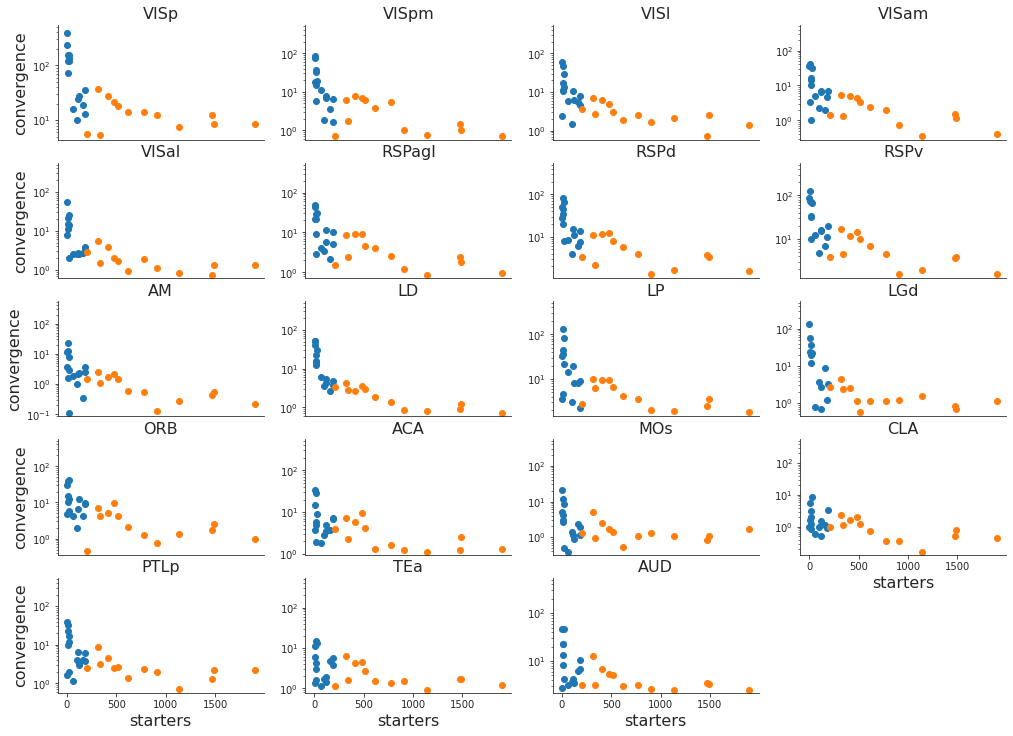

Supplement: S13 Fig — Convergence index vs ns, for ns < 200 (blue) or > 200 (orange). (TIF) [file pone.0278053.s013.tif]

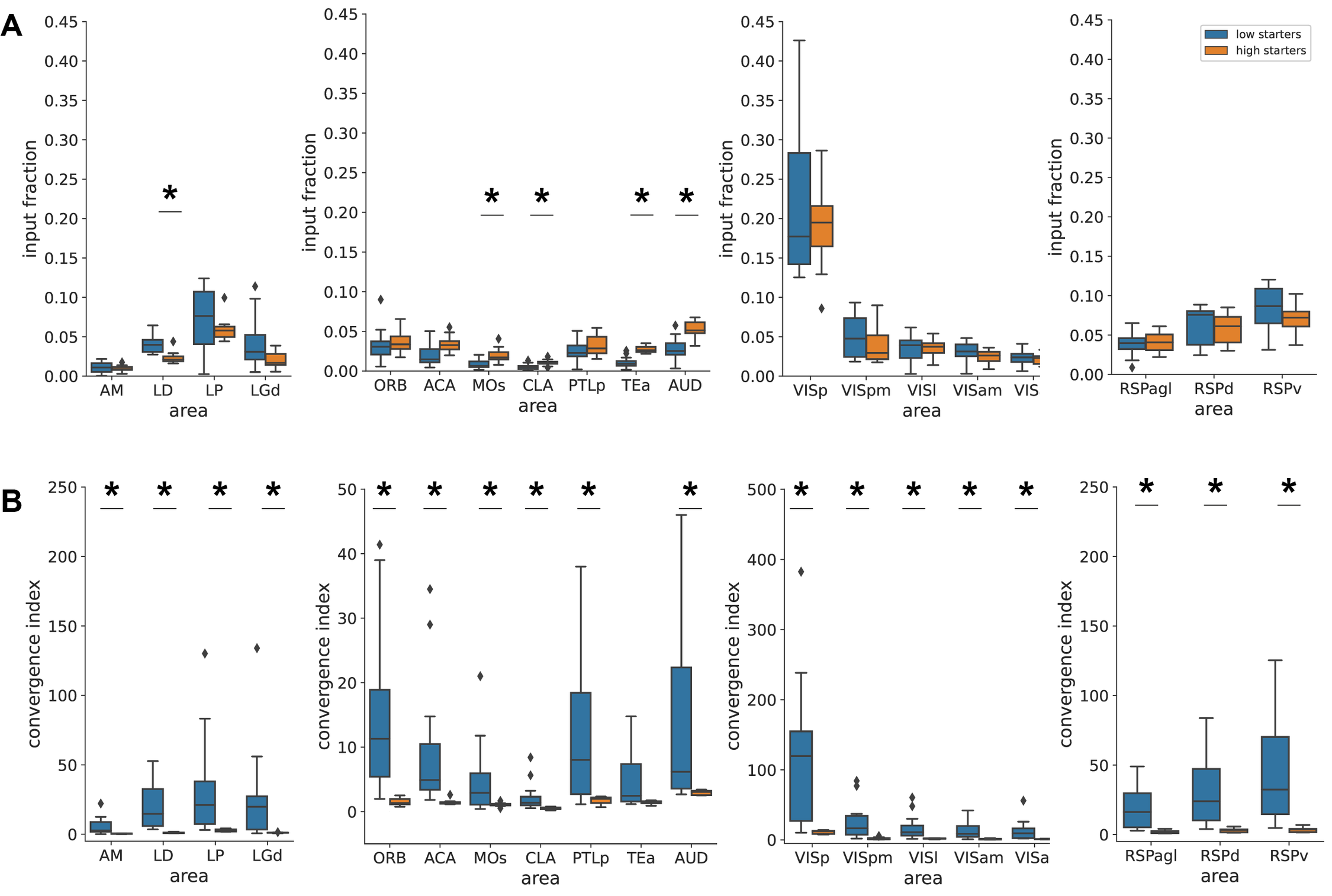

Supplement: S14 Fig — (A) Area input fractions averaged across the low starter range (<125 starters, n = 10, blue) or across the high starter range (>600 starters, n = 10, orange). Statistical differences between area input fraction for low and high ns are indicated by *. Significance was calculated using multiple t-tests corrected for multiple comparisons using the Benjamini-Hochberg method with a false discovery rate of 10%. (B) Same as A, using convergence index per area. (TIF) [file pone.0278053.s014.tif]

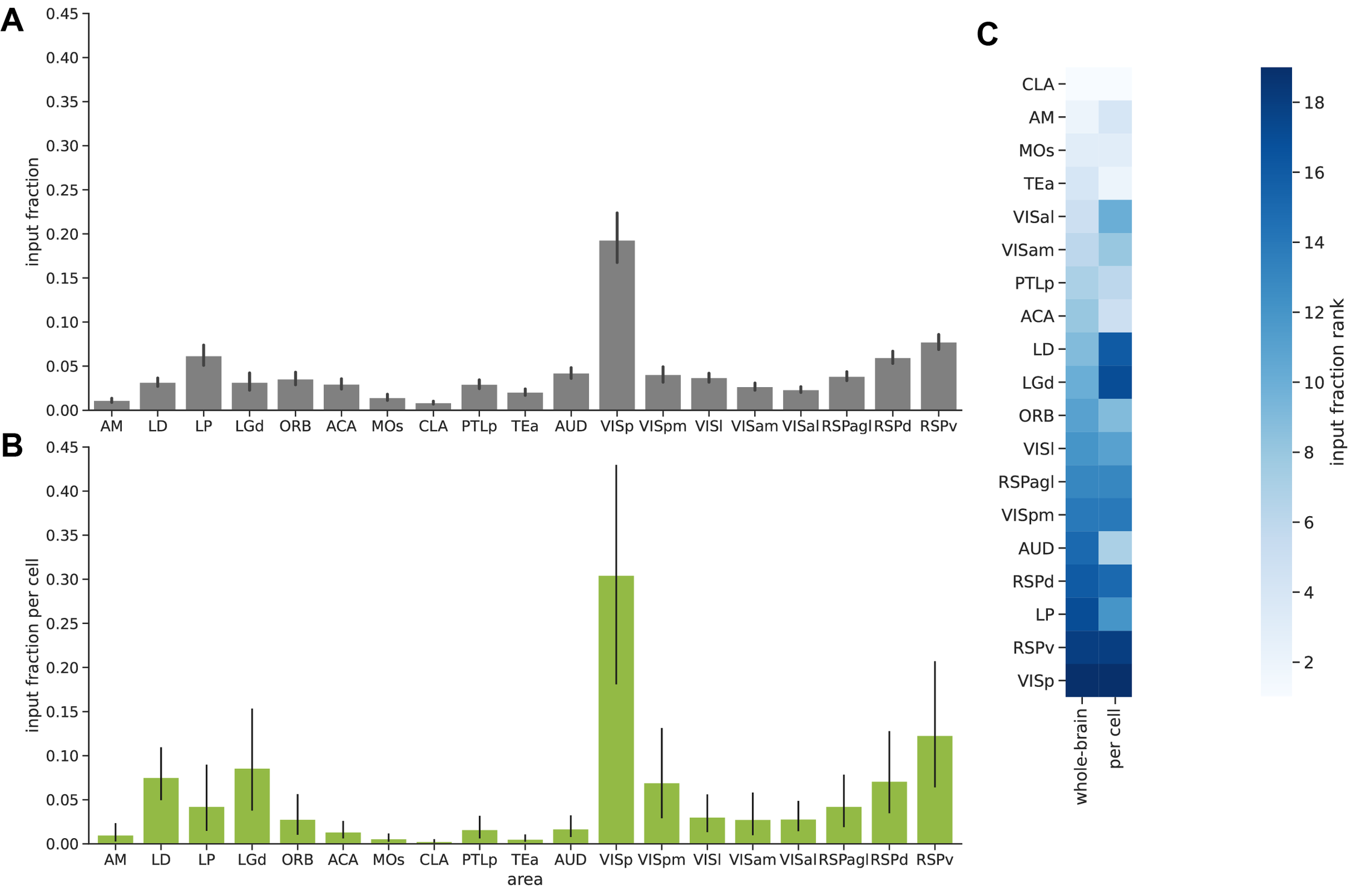

Supplement: S15 Fig — (A) Area input fraction calculated over the full range of starter cells (error bars are s.d.) or (B) calculated from the y-intercept of log(ni) vs log(ns) relationship converted to linear scale (error bars are 95% confidence intervals from residuals bootstrap). (C) Areas ranks obtained via both methods are showed as a heatmap (lowest rank correspond to smallest fraction, lighter colors). (TIF) [file pone.0278053.s015.tif]

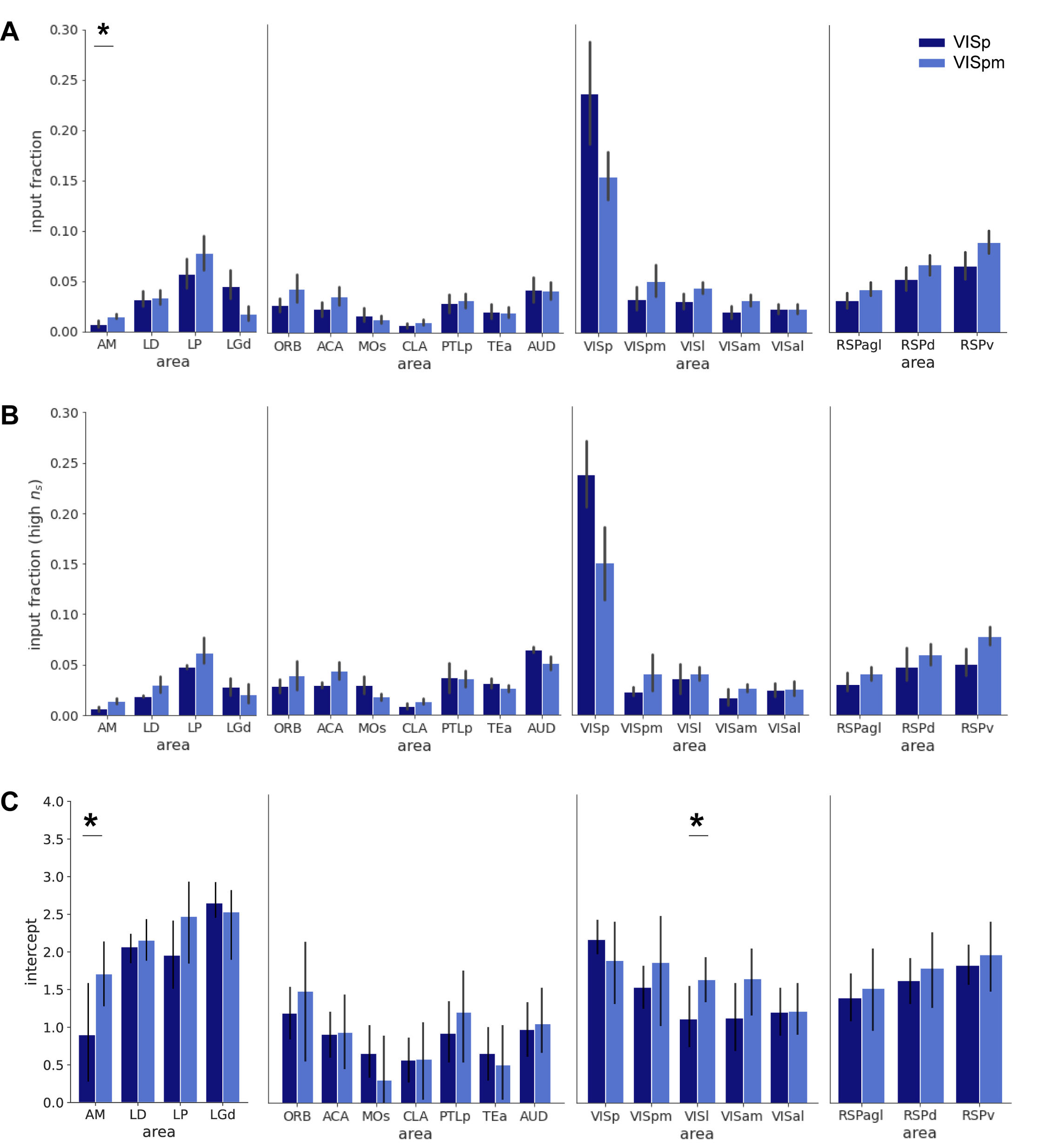

Supplement: S16 Fig — (A) Area input fraction calculated over the full range of starter cells, for experiments with target area in VISp (dark blue) or VISpm (light blue). Asterisks indicate significant difference. Significance is calculated using multiple t-tests and is corrected for multiple comparisons using the Benjamini-Hochberg method with a false discovery rate of 10%. (B) Same as A, but for ns>200. (C) Y-intercept of log-transformed ni vs ns relationship. Significance is assessed by subtracting bootstrapped values of the y-intercepts between target areas. If the resulting distribution does not contain 0, the intercepts are considered significantly different. NB: areas VISp and VISpm act either as local or distal input areas, depending on the starter cells’ location. (TIF) [file pone.0278053.s016.tif]

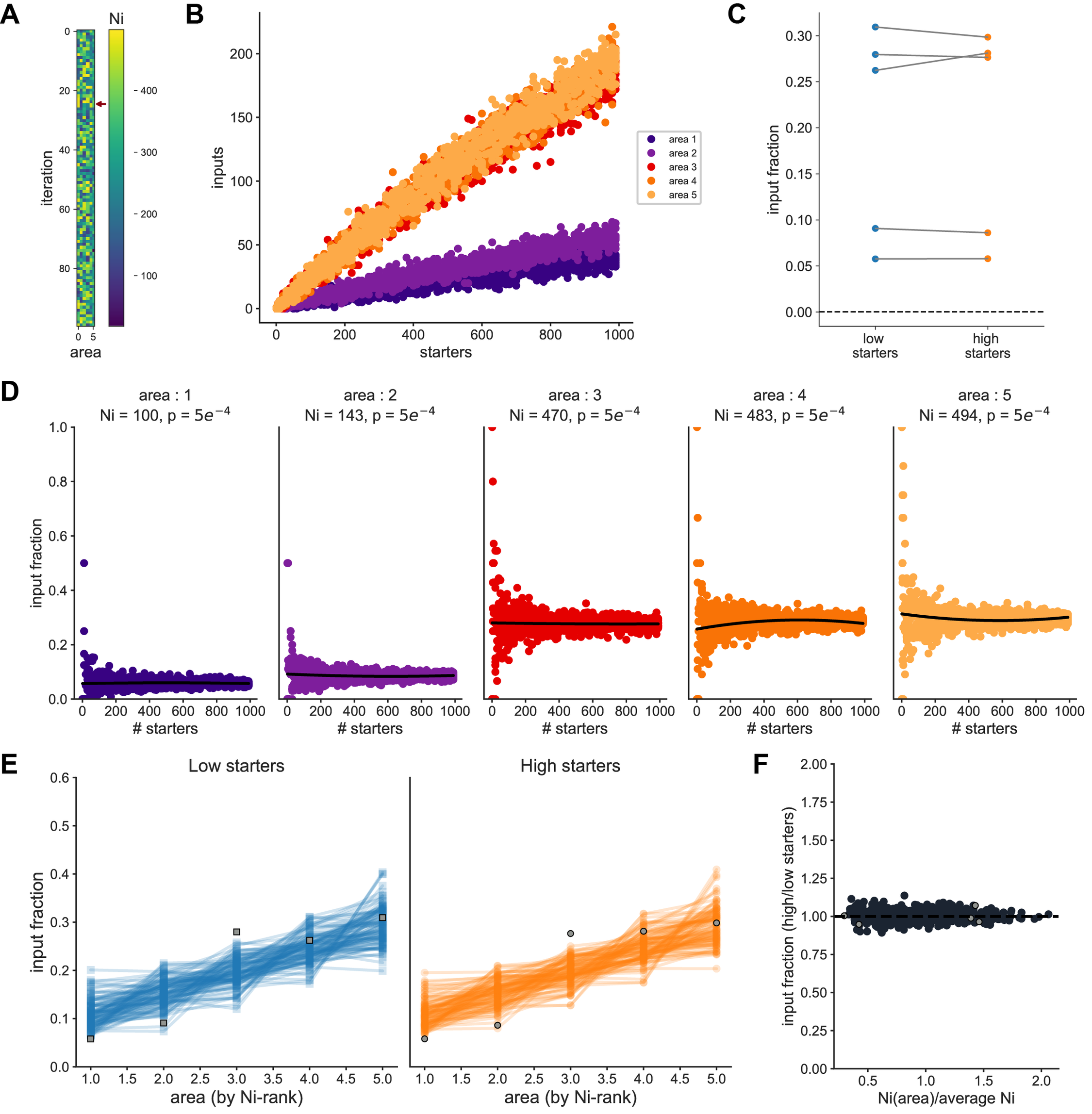

Supplement: S17 Fig — Simulations performed using the probabilistic model with 5 input areas (100 iterations). For each iteration, the connection probability for each input area was p = 5*10−4 and the size of the number of input cells Ni per area was randomly drawn between 100 and 500. (A-F), as in Fig 5. (TIF) [file pone.0278053.s017.tif]

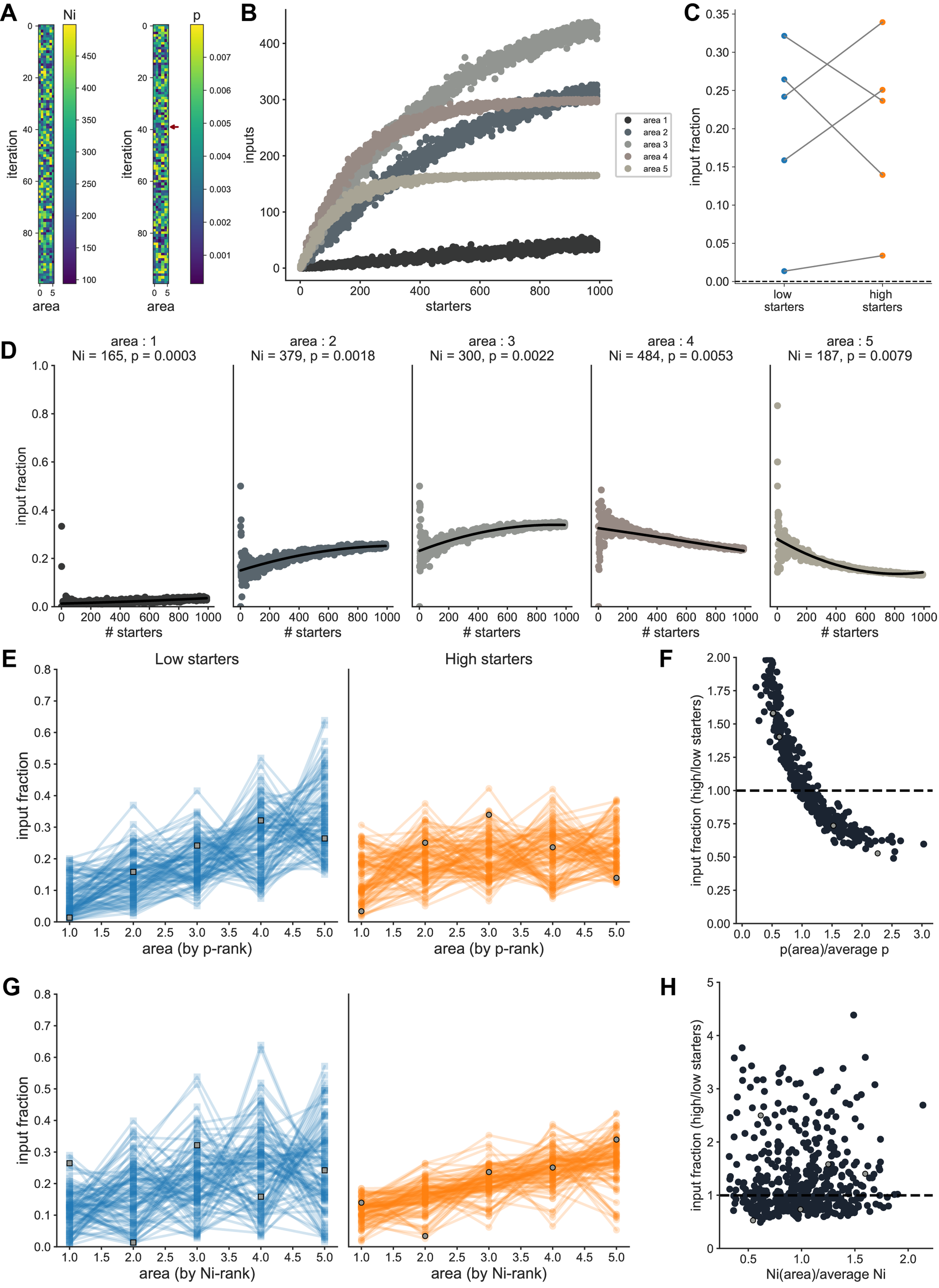

Supplement: S18 Fig — Simulations performed using the probabilistic model with 5 input areas (100 iterations). For each simulation, the connection probability p for each input area was randomly drawn between 1*10−4 and 8*10−3 and the size of the number of input cells Ni per area was randomly drawn between 100 and 500. (A-F), as in Fig 5, (G-H), same as (E-F) but to compare the effect of relative Ni. (TIF) [file pone.0278053.s018.tif]

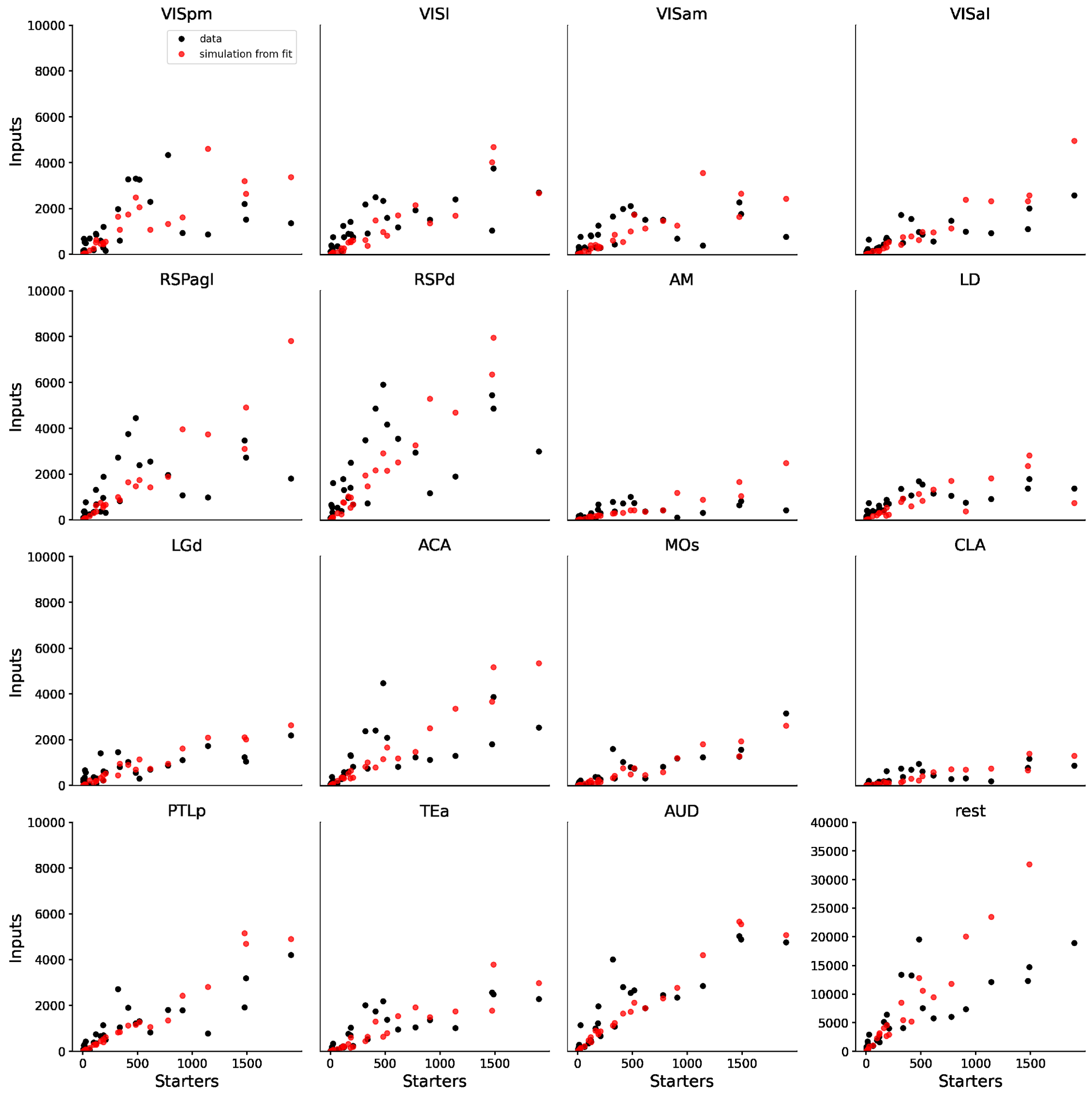

Supplement: S19 Fig — Input vs starters relationships for the data (black) or simulations with parameters obtained from the model fit of the data (red), for one iteration of the fit, for all areas not shown in Fig 6. (TIF) [file pone.0278053.s019.tif]

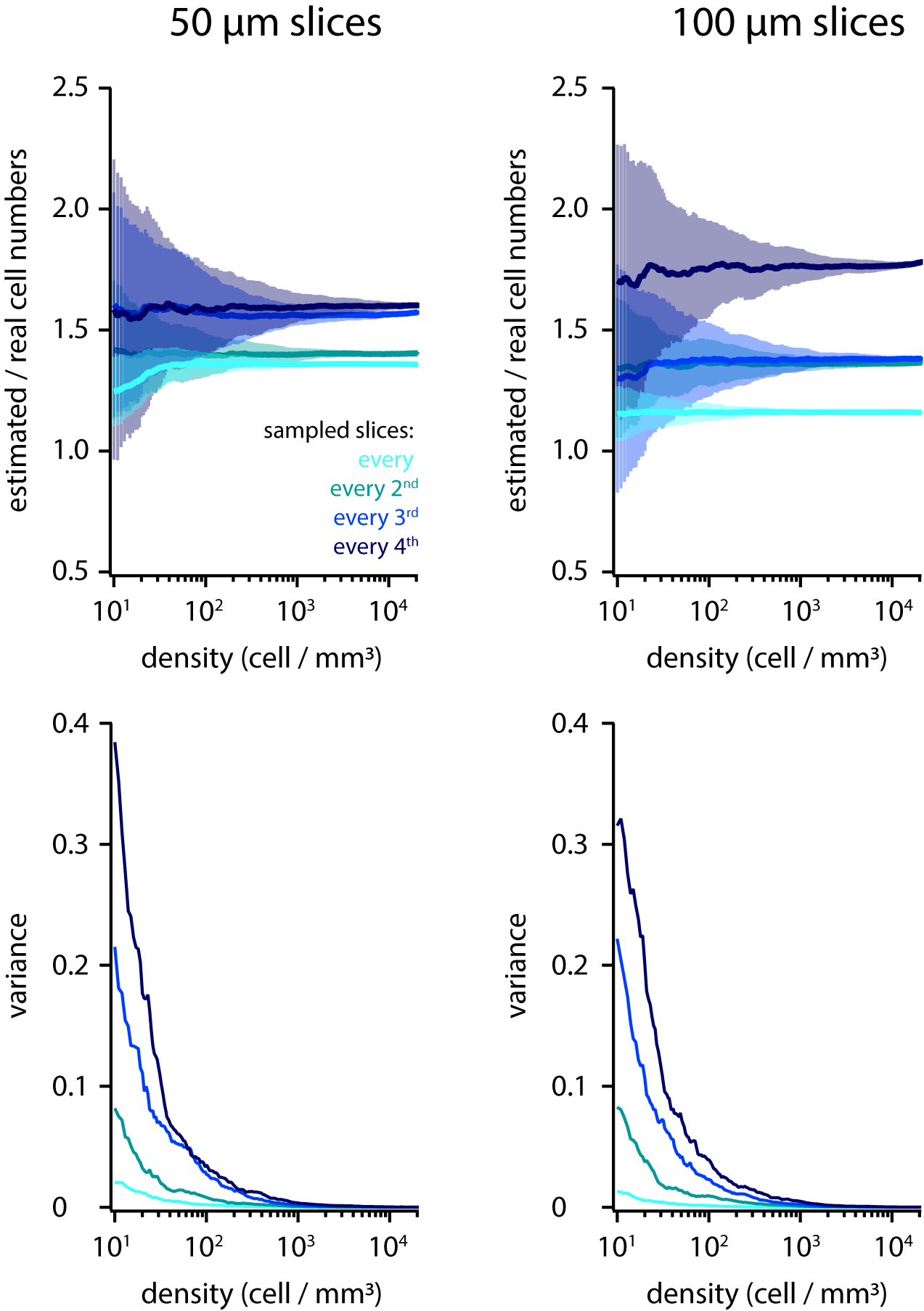

Supplement: S20 Fig — Top: the ratio between cell numbers estimated by counting in sliced tissue and the number of cells in the volume plotted versus cell density. Colours indicate slice sampling. Simulation data for two slice thicknesses, 50 and 100 μm, are shown. Bottom: variance of cell counts versus cell density for different slice sampling values and slice thickness. (TIF) [file pone.0278053.s020.tif]
